# Logistic Entropy of Trabecular Bone Score in SLE Patients and New Type Visualizations

**DOI:** 10.1155/2022/3292249

**Published:** 2022-10-27

**Authors:** Gülsen Kılınç

**Affiliations:** Department of Mathematics and Science Education, Faculty of Education, Adıyaman University, 02040 Adıyaman, Turkey

## Abstract

The medical images and reference intervals, in the diagnosis and treatment process of diseases, are undoubtedly very important. Clear and easy interpretation of images and reference intervals derived from medical devices and statistical methods, respectively, are of great importance for doctors in the diagnosis and treatment process. In this article, for systemic lupus erythematosus disease, we have transformed the reference intervals into fuzzy sets and calculated the entropy values to find the uncertainty contained in the reference interval of systemic lupus erythematosus disease, so the doctors can keep in view patient treatment using entropy values. Finally, for better observations of patients, with the help of the entropy functions, new types of medical images were given for some trabecular bone diseases using Wolfram Mathematica 7.0. We should note that this type of medical images for bone mineral density is not in medicine.

## 1. Introduction

In the diagnosis of diseases, physical examination findings of the patient, preliminary information, and a series of laboratory tests and imaging methods are used. The results obtained are evaluated, and the treatment method is determined. There are many devices used for diagnosis in medicine. Some of them are ECG, EEG, and DXA. While ECG is a device used for recording the electrical activity that occurs during the contraction of the heart graphically, EEG is a device used to measure the electrical activity in the human brain. Graphical images that give the amplitudes and durations of these signals in the ECG or EEG observed by doctors are used to predict diseases in the human body. The device DXA gives a numerical value for bone mineral density.

In both medicine and veterinary, it is of great importance for physicians to be able to accurately read the data of all medical diagnostic tools such as ECG, EEG, and DXA devices, for accurate diagnosis and treatment. A long time may be spent interpreting this data and even a small mistake can make a big difference in the diagnostic process; also, in case of young (inexperienced) doctors and veterinarians, some important data may be overlooked. Sometimes, data in a medical device may not contain complete and accurate information about the patient's disease. Consequently, lots of problems will occur due to the lack of doctor's experience and the lack of data in medical device. This means that it is necessary to give full and reliable information to doctors.

The most important questions to ask about graphical records and reference intervals are as follows:

- Do medical chart records and reference intervals provide complete and accurate information about the diseases they represent?
- Could reference intervals have a lack of knowledge?

The best answer to these questions can be given by using the concept of entropy discussed in fuzzy set theory which is the subject of this article.

Let us note that “*Everything in medicine is fuzzy*” (see [1], 17]).

The fuzzy set theory is used to develop mathematical models in different technical fields and makes an important and increasing contribution to medical research. One of the earliest models discussed by other scientists in medicine was created by Sanchez [2, 3]. This created model provided some answers to questions about diagnostic selection. Selection should be on the basis of clinical symptoms alone, assuming symptoms are typical for all diagnoses considered.

In this article, since our goal is to measure the accuracy of information in medical reference intervals, we will discuss the entropy of medical reference intervals, where medical intervals relate to bone density in SLE disease. We know that, in medicine, the majority of measurements of body values are denoted with of help numerical (reference) intervals. Towards the endpoints of these reference intervals, it becomes very difficult to make a decision about the patient's illness. For instance, bone density is determined by the DXA device. If someone's *T*-score is in the range of [−2.5, −1.0], it means that he or she has low bone density or osteopenia. The *T*-score is an indicator of how much one's bone mass differs from an average healthy 30-year-old adult [4]. The *T*-score of normal bone mineral density (BMD) for a person should be in the range of [−1, 1].

It is known that people who have a score in this range do not typically need treatment, but it is useful for them to take steps to prevent bone loss, such as having adequate amounts of calcium and vitamin D and doing weightlifting exercise, etc. [5].

Do medical reference intervals such as [−1, 1], [−2.5, −1.0] always contain correct complete information? The answers to this and similar questions will be given below.

Recently, Şengönül et al. [6, 7] have also made some investigations using the concepts of fuzzy set and entropy.

Before moving on to the main topic of this article, we will summarize some background information on fuzzy sets and entropy of the fuzzy sets. Then, we will do some calculations but these calculations are completely different from those of Czogala and Leski [8].

## 2. Some Basic Knowledge about Fuzzy Sets

Zadeh [9] defines fuzzy sets as a class of continuous membership degrees. A fuzzy set in universe of discourse *X* is described by a membership function *A* which assosicates with each elements of *X* a real number *A*(*x*) in [0, 1].*A*(*x*) is the membership grade of *x* in fuzzy set *A*. We demonstrate by *ℱ*(X) the collection of all fuzzy subsets of *X*. The possibility of the fuzzy set to express gradual transitions from membership to nonmembership and vice versa has a wide range of uses. It provides us not only with a meaningful and powerful representation of measurement uncertainties but also a meaningful representation of uncertain concepts expressed in natural language. The characteristic function of a set assigns a value of 1 or 0 to each individual in the universal set, thus distinguishing between members and nonmembers of the studied set. This function ensures that the values assigned to the elements of the universal set remain within a certain range. It also shows the degree of membership of these elements in that set. Larger values indicate higher degrees of cluster membership. Such a function is called a membership function and the set it defines is called a fuzzy set. Let us define fuzzy set *A* on the set ℝ with membership function as follows:

(1) $$\begin{array}{c} A\left(x\right)=\left\{\begin{array}{cc} \frac{h_{A}}{u_{1}-u_{0}}\left(x-u_{0}\right), & x\in \left[u_{0},u_{1}\right),\\ \frac{-h_{A}}{u_{2}-u_{1}}\left(x-u_{1}\right)+h_{A}, & x\in \left[u_{1},u_{2}\right],\\ 0, & \text{others}. \end{array}\right. \end{array}$$

The notation *h*_*A*_ denotes the height of the fuzzy sets *A*, where *u*_0_, *u*_1_, *u*_2_ ∈ ℝ and *u*_0_ ≤ *u*_1_ ≤ *u*_2_. For brief, we write triple (*u*_0_, *u*_1_ : *h*_*A*_, *u*_2_) for fuzzy set *A*. The notation *ℱ* denotes the set of the all fuzzy sets in the form *u* = (*u*_0_, *u*_1_ : *h*_*A*_, *u*_2_) on the ℝ.

In the fuzzy set theory, it is known that the fuzziness of a fuzzy set is an important matter and there are many methods to measure the fuzziness of a fuzzy set. At first, the fuzziness was thought to be the distance between the fuzzy set and its nearest nonfuzzy set. Later, the entropy was used instead of fuzziness [10, 11]. Well, then, what is the entropy? These definitions are given in [6, 7] but here it will be summarized.

Let *A* be a fuzzy set and *A*(*x*) be the membership function of *A*. The function *H* which has the following properties is called the entropy of *A*:

- For the crisp set *A* in *X*, *H*(*A*) is zero
- For all *x* ∈ *A*, if *A*(*x*) = 1/2, *H*(*A*) is single maximum value
- For a fuzzy set *A*, *H*(*A*^*c*^) and *H*(*A*) are equal, where *A*^*c*^ is the complement of *A*
- For the fuzzy sets *A*, *B*, if *B*(*x*) ≤ *A*(*x*), for *A*(*x*) ≤ 1/2 and *B*(*x*) ≥ *A*(*x*)

for *A*(*x*) ≥ 1/2 then *H*(*A*) ≥ *H*(*B*).

The *h* function defined from [0, 1] to [0, 1] satisfying the following conditions is called the entropy function.

Monotonically increasing at [0, 1/2] and decreasing at [1/2, 1].

*h*(*x*) = 0 if *x* = 0 and *h*(*x*) = 1, if *x* = 1/2.

The equality *H*(*A*(*x*)) = *h*(*A*(*x*)) is provided between the *H* and *h* functions at a fixed element *x*.

Here are some well-known entropy functions:

(2) $$\begin{array}{c} h_{1}\left(x\right)=4x\left(1-x\right),\\ h_{2}\left(x\right)=-x\ln x-\left(1-x\right)\ln\left(1-x\right),\\ h_{3}\left(x\right)=\min\left\{2x,2-2x\right\}\\ h_{4}\left(x\right)=\left\{\begin{array}{cc} 2x, & x\in \left[0,\frac{1}{2}\right],\\ 2\left(1-x\right), & x\in \left[\frac{1}{2},1\right]. \end{array}\right. \end{array}$$

Note that the function *h*_1_ is the logistic function, *h*_2_ is called Shannon function, and *h*_3_ is the tent function.

Let *X* be a continuous universal set, *A* is fuzzy set on, *u*(*x*) be membership function of *A*, and *h* ∈ {*h*_1_, *h*_2_, *h*_3_}. Then, the total entropy of the fuzzy set *A* on the *X* is defined as below:

(3) $$\begin{array}{c} \omega_{A}=\int_{x\in X}^{.}h\left(u\left(x\right)\right)p\left(x\right)dx, \end{array}$$

where *p*(*x*) is the probability density function of the available data in *X*. Let ∘ be the usual composite operation and *h*(*u*(*x*)) = *h*∘*u*(*x*) [12]. If we take *p*(*x*) = 1 and *h* = *h*_1_ in the (3) then *ω*_*A*_ is called logistic entropy of the fuzzy set *A*.

It is known that the value of *ω*_*A*_ depends on support of the fuzzy set *A*. If *A* is a fuzzy set on the set *R* with membership function (1) then we see that the logistic entropy of fuzzy set *A* is equal to

(4) $$\begin{array}{c} \omega_{A}=c\left(2h_{A}-\frac{4}{3}h_{A}^{2}\right)l\left(A\right), \end{array}$$

for *p*(*x*) = *c*, (*c* = cons.) and *h* = *h*_1_, where $l\left(A\right)=\sup\left\{\left(x-y\right)\text{: }x,y\in \bar{\left\{x\in R:A\left(x\right)>0\right\}}\right\}$ [6].

For any two fuzzy sets *A* and *B*, if *ω*_*A*_ ≥ *ω*_*B*_ then the fuzzy set *B* is crisp than *A*.

The consistency of an interval obtained by statistical methods with disease can be determined with an entropy degree. When the given range is converted to a fuzzy set, if the calculated entropy value is less than 0.5, the range is compatible with the disease. If the entropy value is greater than 0.5, then the range given by statistical methods may not be compatible with the disease. If the entropy value is greater than 1, the range is not secure.

## 3. Materials and Methods

It is known that, in medicine, reference intervals are obtained by statistical methods and include disease-related data. However, the accuracy of the information contained in these reference intervals are very important. In this article, the reference intervals have been collected from literature so the database have been created for disease SLE and other bone mineral density (see [13–15]). These data formed our materials. As methods, we transformed the reference intervals into fuzzy sets and calculated the entropy values to find the uncertainty contained in the reference intervals. After the entropy values have been computed using by reference intervals for normal bone density and SLE. So, the doctors can monitor patient treatment using entropy values. Finally, for better observations of patients, new types of medical images were obtained for some trabecular bone diseases using Wolfram Mathematica 7.0.

## 4. Background and Entropy Computations for Trabecular Bone Score

Trabecular bone score (TBS) is a computer software application installed on DXA machines. The program takes the DXA image of the lumbar spine (low back) and creates a grayscale pixel image of the vertebral trabecular bone microstructure. The resulting image provides an indirect measure of the trabecular microarchitecture. A dense structure, with lots of well-connected trabeculae, has lots of pixels with small amplitude changes. They are variations of so light and light gray. Think of it like a dense sponge with very small holes [16]. A high trabecular bone score means that the bone microarchitecture is dense and well-connected. Conversely, a low trabecular bone score means that bone microarchitecture is incomplete and weakened.

In a study performed by Shafiee et al. [15] on 691 participants (aged ≥18 years, 381 men and 310 women), the mean and standard deflection (SD) of the TBS value for men were 1.420 ∓ 0.094 and the age at the peak TBS was 30.0 years. For women, this value corresponds to 1.428 ∓ 0.070 and the age at the peak TBS was 24.5 years. Also, it has been decided two SDs below the mean of TBS were 1.326 in men and 1.357 in women.

Further, they have proposed normal range for TBS values ≥ 1.326 according to men and this TBS is considered to be normal. If the value is in the interval [1.231, 1.326], it is considered to be partially degraded microarchitecture; and if TBS ≤ 1.231, this situation is defined as degraded microarchitecture.

Among women, TBS categories are defined as normal, if the value ≥1.357; as partially degraded, if the value is in the interval [1.287, 1.357], and as degraded, if the value ≤ 1.287.

*ω*_N_(TBS), *ω*_PD_(TBS), and *ω*_FD_(TBS) denote the entropies of membership functions of normal trabecular bone system, partially degraded trabecular bone system, and full degraded trabecular bone system, respectively, and membership functions are determined with (1).

Basing on data given by Shafiee et al. in [15], *ω*_N_(TBS), *ω*_PD_(TBS), and *ω*_FD_(TBS) are calculated as follows with *p*(*x*) = 1.

**Figure 1:**
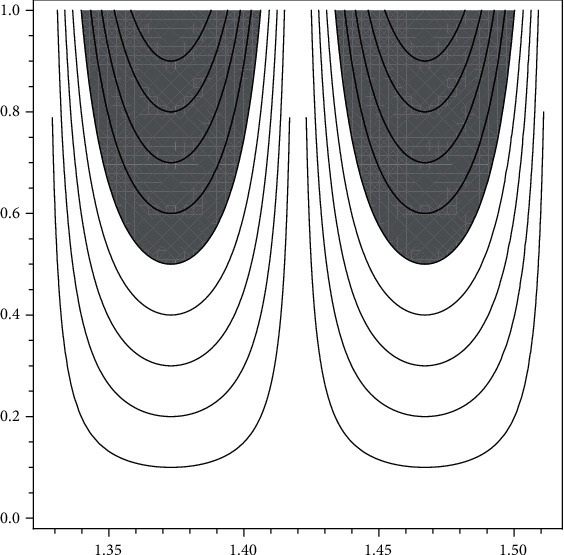
New type visualizations of normal trabecular bones for men according to data of [15].

The entropy of partially degraded microarchitecture of trabecular bone system *ω*_N_(TBS) is

(5) $$\begin{array}{c} \omega_{\text{N}}\left(\text{TBS}\right)=\int_{x\in \left[1.326,1.420\right]}^{.}h_{1}\left(u_{\text{N}}\left(\text{TBS}\right)\left(x\right)\right)p\left(x\right)dx+\int_{x\in \left[1.420,1.514\right]}^{.}h_{1}\left(u_{\text{N}}\left(\text{TBS}\right)\left(x\right)\right)p\left(x\right)dx=0.125333, \end{array}$$

(see Figure 1 for graphical representation).

**Figure 2:**
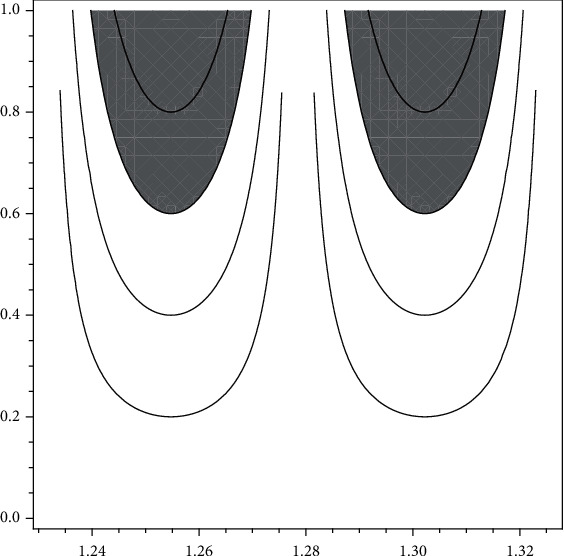
New type visualizations of partial degraded microarchitecture at trabecular bones for men according to data of [15].

The entropy of partially degraded microarchitecture of the trabecular bone system *ω*_PD_(TBS) is

(6) $$\begin{array}{c} \omega_{\text{PD}}\left(\text{TBS}\right)=\int_{x\in \left[1.231,1.2785\right]}^{.}h_{1}\left(u_{\text{PD}}\left(\text{TBS}\right)\left(x\right)\right)p\left(x\right)dx+\int_{x\in \left[1.2785,1.326\right]}^{.}h_{1}\left(u_{\text{PD}}\left(\text{TBS}\right)\left(x\right)\right)p\left(x\right)dx=0.0633, \end{array}$$

(see Figure 2 for graphical representation).

**Figure 3:**
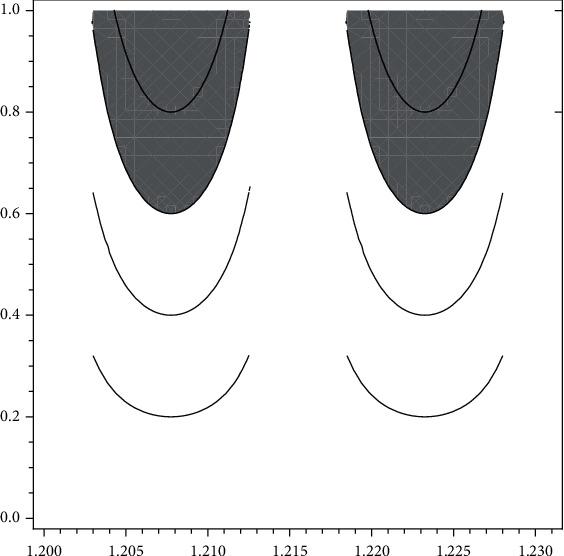
New type visualizations full degraded microarchitecture at trabecular bones for men according to data of [15].

Please see Figure 3.

It is known that if entropy is 0 then the information (data) is crisp. But we find that *ω*_N_(TBS) = 0.125333 > 0. This indicates that TBS ≥ 1.326 reference range contains uncertainty for men and the data obtained from DXA devices is not very reliable.

Likewise, the reference interval [1.231, 1.326] does not give enough accurate information, because of *ω*_PD_(TBS) = 0.0633 > 0. But this entropy value is small from the value of *ω*_N_(TBS). Since *ω*_PD_(TBS) = 0.0633 < *ω*_N_(TBS) = 0.125333, the *ω*_PD_(TBS) is crisp than *ω*_N_(TBS). In other words, *ω*_PD_(TBS) contains more accurate information than *ω*_N_(TBS). Then, the reference interval [1.231, 1.326] includes more right knowledge. Thus, again the medics should be careful in their decisions. Similarly, the same comments apply to each of the following cases:

The entropy of full degraded micro architecture of trabecular bone system *ω*_FD_(TBS) is

(7) $$\begin{array}{c} \omega_{\text{FD}}\left(\text{TBS}\right)=\int_{X\in \left[1.2,1.2155\right]}^{.}h_{1}\left(u_{\text{FD}}\left(\text{TBS}\right)\left(x\right)\right)p\left(x\right)dx+\int_{x\in \left[1.2155,1.231\right]}^{.}h_{1}\left(u_{\text{FD}}\left(\text{TBS}\right)\left(x\right)\right)p\left(x\right)dx=0.0206, \end{array}$$

(see Figure 3 for graphical representation).

The similar computations for women can be obtained with same way, so we omit it.

**Figure 4:**
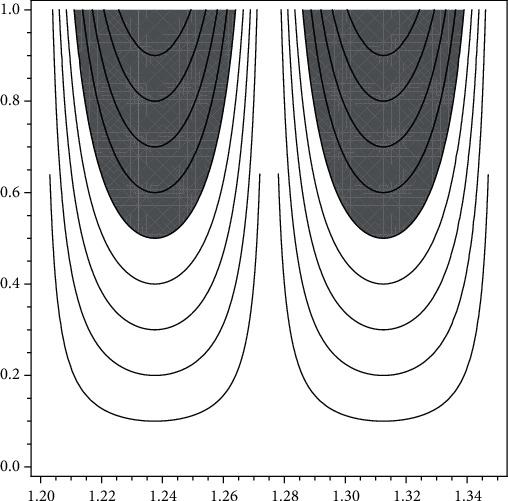
New type visualizations of partial degraded microarchitecture at trabecular bones for postmenopausal women according to data of [14].

In another study by Shevroja et al. [14], it was determined that if TBS ∈ [1.350, −[ , this score is normal BMD at trabecular bone locations in postmenopausal women. If TBS ∈ [1.200,1.350], this score is considered consistent with partially degraded microarchitecture and for values of TBS less than 1.200, the microarchitecture is degraded [14]. Let us suppose that *p*(*x*) = 1. In this case, according to Shevroja et al., the entropy of the trabecular bone score for partial degraded microarchitecture *ω*_PD_(TBS) is

(8) $$\begin{array}{c} \omega_{\text{PD}}\left(\text{TBS}\right)=\int_{X\in \left[1.2,1.275\right]}^{.}h_{1}\left(u_{\text{PD}}\left(\text{TBS}\right)\left(x\right)\right)p\left(x\right)dx+\int_{x\in \left[1.275,1.35\right]}^{.}h_{1}\left(u_{\text{PD}}\left(\text{TBS}\right)\left(x\right)\right)p\left(x\right)dx=0.1, \end{array}$$

(see Figure 4 for graphical representation).

## 5. The Background and Entropy Computations for Systemic Lupus Erythematosus

SLE, short for systemic lupus erythematosus disease, is the most common type of lupus. SLE is an autoimmune disease in which the immune system attacks its own tissues, causing widespread inflammation and tissue damage in the affected organs. It can affect the joints, skin, brain, lungs, kidneys, and blood vessels, and no complete cure has yet been found for lupus. But the disease can be controlled through medical support and lifestyle [17]. Some of the symptoms of SLE are a vague feeling of discomfort, fatigue, skin rashes, fever, pain or swelling in the joints and muscles, loss of appetite, weight loss, and skin problems. Skin problems are common in SLE [18]. Studies show increased bone loss and fracture in individuals with SLE. People with lupus are also at a very high risk of osteoporosis. Glucocorticoid drugs used to treat SLE can also trigger significant bone loss. Furthermore, inactivity due to pain and fatigue also increases the risk of osteoporosis. Studies also show that bone loss in lupus may occur as a direct result of the disease. What is worrying is that 90 percent of people affected by lupus are women, a group already at high risk for osteoporosis [19]. Also, it is known that in patients with normal or osteopenic bone density who have had minimal trauma fractures, the trabecular bone score (TBS) can provide a measure of bone quality. The studies carried out bone mass measurement at several sites showed a significant difference between patients coming for control and SLE patients. Patients with SLE were generally characterized by a loss in BMD at the lumbar vertebra and proximal left femur. It is known that none of the SLE patients had a BMD reading below the theoretical fracture threshold 0.812 gr∕cm^2^, but if bone loss persisted, SLE patients seemed more likely to develop fractures of the lumbar spine [20]. Some of the very recent studies on bone density in this disease include [21–25].

**Figure 5:**
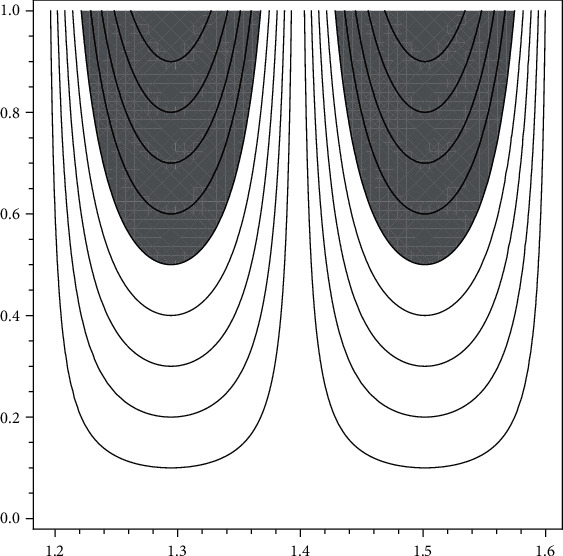
New type visualizations non-SLE patients at lumbar spine for postmenopausal females according to data of [13].

In [13], Ruaro et al. studied a comparison between bone involvement in SLE patients and healthy matched subjects and it is seen that in a total of 40 SLE females (average age 54.1 ∓ 16.3) years), the lumbar spine TBS score was statistically significantly lower in SLE patients (range 0.797 ± 0.825) than non-SLE (range 1.398 ± 0.207) in all areas examined. Another claim of Ruaro et al. is that SLE is associated with significant low bone mass as evidenced by DXA and TBS. In this case, the entropy of lumber spine TBS score of females (average age 54.1 ± 16.3 years) with non-SLE *ω*_NSLE_(TBS) is

(9) $$\begin{array}{c} \omega_{\text{NSLE}}\left(\text{TBS}\right)=\int_{X\in \left[1.191,1.398\right]}^{.}h_{1}\left(u\left(\text{TBS}\right)\left(x\right)\right)p\left(x\right)dx+\int_{x\in \left[1.398,1.605\right]}^{.}h_{1}\left(u\left(\text{TBS}\right)\left(x\right)\right)p\left(x\right)dx=0.276, \end{array}$$

(see Figure 5 for graphical representation).

**Figure 6:**
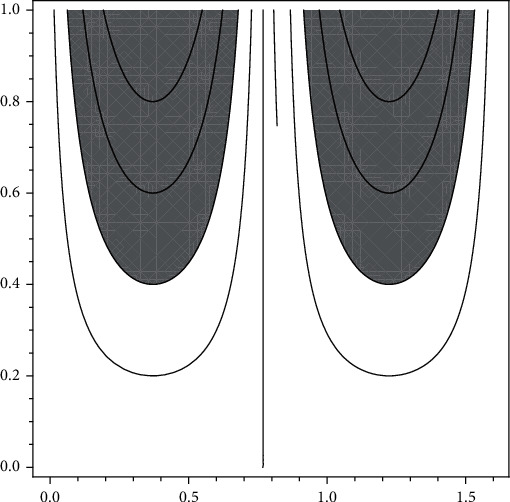
New type visualizations SLE patients at lumbar spine for postmenopausal females according to data of [13].

Entropy of the lumbar spine TBS score of females with SLE *ω*_SLE_(TBS) is

(10) $$\begin{array}{c} \omega_{\text{SLE}}\left(\text{TBS}\right)=\int_{X\in \left[-0.028,0.825\right]}^{.}h_{1}\left(u\left(\text{TBS}\right)\left(x\right)\right)p\left(x\right)dx+\int_{x\in \left[0.825,1.622\right]}^{.}h_{1}\left(u\left(\text{TBS}\right)\left(x\right)\right)p\left(x\right)dx=1.05443, \end{array}$$

(see Figure 6 for graphical representation).

## 6. Conclusions

In this study, we have collected data about trabecular bones in literature for the normal, partial degraded, and full degraded lumbar spine or femur neck. Then, on reference intervals, we have constructed membership functions for normal, partial degraded, and full degraded trabecular bones and calculated entropy values for these trabecular bones using the logistic entropy function. It was seen that

- The entropy values are different for every bone illness phase
- Also, these differences are seen in new type visualizations which they have given in the text
- Most importantly, since there are large entropy values in reference intervals, physicians must be very careful if the disease is to be decided using reference intervals
- It is known that if entropy is 0 then the information (data) is crisp. In study, it is found that *ω*_N_(TBS) = 0.125333 > 0. This indicates that the TBS ≥ 1.326 reference interval contains uncertainty for men, and the data obtained from DXA devices is not very reliable
- Since *ω*_PD_(TBS) = 0.0633 < *ω*_N_(TBS) = 0.125333, *ω*_PD_(TBS) is crisp than *ω*_N_(TBS)

## Data Availability

The data supporting this article were obtained by the authors from the relevant literature.
